# Nursing students perspectives of psychosocial care: cross-sectional study

**DOI:** 10.1186/s12912-023-01548-7

**Published:** 2023-10-19

**Authors:** Ahmad H. Al-Nawafleh, Falah Zaal Altarawneh

**Affiliations:** 1 Department of Adult Health Nursing, Faculty of Nursing, Mutah University, Mutah, 61710 Jordan; 2 Falah Zaal Altarawneh (MSN, RN), Department of Community Health, Faculty of Nursing, Mutah University, Mutah, 61710 Jordan

**Keywords:** Psychosocial Care, Nursing Care, Nursing Students, Communication Skills, Education Process, Emotional Support

## Abstract

**Background:**

Psychosocial care is an integral component of holistic nursing practices. This study aimed to examine how fourth-year nursing students at Mutah University perceive various care characteristics, specifically psychosocial care.

**Methods:**

A quantitative cross-sectional study was conducted using the Caring Dimension Inventory (CDI). Data were obtained from 105 nursing students before graduating from a Jordanian public educational institution. Data analyzed using the scoring of CDI and descriptive statistics.

**Results:**

The mean scores in the professional and technical domain of care were statistically higher for fourth-year nursing students (4.69 ± 0.25). This exceeded their scores in the psychosocial domain of care (3.37 ± 1.20). This suggests that fourth-year nursing students prioritize professional and technical care over psychosocial care.

**Conclusions:**

Fourth-year nursing students tended to perceive the technical aspects of care as more significant than the psychosocial aspects. This highlights the need for nursing schools and healthcare providers to reconsider their focus and prioritize the importance of psychosocial care.

## Introduction

Psychosocial care is an essential aspect of nursing practice and its significance has been emphasized in various nursing frameworks and guidelines. The International Council of Nurses (ICN) has identified psychosocial care as a key component of comprehensive nursing care and is essential for universal health coverage [1]. The American Nurses Association (ANA) has also emphasized the importance of psychosocial care in nursing practice and recommends that nurses provide holistic care that addresses the physical, psychological, and social needs of patients [2]. The definition of psychosocial care focuses on the psychological and emotional health. Covers a wide range of topics, including self-esteem, understanding and coping with illness, communication, and social interaction [3]. Although holistic nursing care requires addressing physical and psychosocial needs for better patient outcomes, there is evidence that nurses often do not approach this aspect of care adequately for many reasons [3–8].

As nurses progress in their education, they acquire knowledge, abilities, and attitudes related to psychosocial care. Nursing students play a crucial role in providing psychosocial care to patients. However, research has indicated that nursing students may lack the skills and knowledge needed to provide patients with adequate psychosocial treatment [9–14]. To allow nursing students to provide patients with better care, it is necessary to assess their degree of psychosocial support and pinpoint areas of development [14].

There is a need to understand how many nursing students in the Middle East and particularly in Jordan are prepared to support and provide care based on the psychosocial domain. In Jordan, 9103 nursing students enrolled in seven public and eight private institutions during the year 2021/2022 [15]. Mutah University is recognized as one of the leading schools in Jordan, with 640 students enrolled in regular, parallel, bridging, and international nursing programs. The nursing program at Mutah University features specialized courses, such as mental health nursing, community health nursing, and nursing leadership, with a strong emphasis on addressing the psychosocial dimensions of nursing care. A recent evaluation of the program and a study indicated the need to evaluate the psychosocial competency of nursing students [16].

This article presents the results of a research study conducted at Mutah University, which aimed to investigate the level of psychosocial support available to fourth-year nursing students. The central focus of this study revolved around the following research question: To what extent do fourth-year nursing students at Mutah University receive adequate psychosocial care?

### Literature review

It is widely acknowledged that nursing is a caring profession and that caring is fundamental to professional nursing practice [13, 17–20]. The process by which a nurse responds to a patient as an individual, senses their feelings, and distinguishes them from the norm is frequently described as caring [13, 19]. On the other hand, caring is difficult to define, most likely because it is not limited to nursing [21, 22]. Caring is a primary way of being in a world that is crucial in our relationships with others as well as ourselves [7]. However, as a generally recognized notion, care is at the heart of nursing's scope of practice [22].

The caring science-based curriculum has emerged as the main concept in nursing education. The objective is to teach nursing students how to provide medical care to licensed nurses [7]. Medical and nursing students experience emotional and practical implications of their care [16, 23, 24]. Nursing students must have emotional maturity, sensitivity, dedication, skills, self-assurance, and a sense of duty to their patients [16]. Similarly to what is expected of professional medical and nursing students, they should demonstrate good communication, patience, courage, and supportive attributes in terms of practical skills [10]. Nursing students participate in their educational journey both in the classroom and in real world settings, as these encounters are crucial to their intellectual development and professional advancement [24].

In an environment of intensive international scrutiny of healthcare, nurse educators work to increase professionalism in providing psychosocial care to their students [10]. Although this objective is widely sought, limited research has explored changes in students' perceptions of their own psychosocial care and that provided by their peers throughout their nursing programs. Therefore, the primary objective of this study was to examine the perceptions of final-year nursing students regarding the attributes of psychosocial care within their nursing education.

### Research questions

The following questions were addressed in this study.

1. Which domain of care is perceived as more important by fourth-year nursing students?
2. How do fourth-year nursing students perceive psychosocial care?

## Methodology

### Design

A quantitative design based on a cross-sectional study was used.

### Study population

Bachelors’ nurses were the target of this study. Fourth-year nursing students (n= 138) from Mutah University were invited to participate in this study.

### Study instrument

Participant responses were collected using the CDI. This questionnaire, established by Watson in 1997, assesses how people view their compassion. Comprising 25 items, the CDI covers aspects such as technical and professional care, inappropriate selflessness, acceptable selflessness or altruism, and psychological care [20].

The inventory was used to collect data on nursing students’ perspectives on various aspects of nursing care. The Likert scale was used, ranging from strongly disagree to strongly agree on a five-point scale. In this study, the assessment was intended to gain insight into students' perceptions of care with a specific emphasis on psychosocial care.

### Reliability and validity

The researchers utilized the Arabic version of the tool, which was validated and found to be reliable within the Jordanian cultural context, achieving a Cronbach’s alpha coefficient of 0.89 [20]. Furthermore, we assessed the dependability, measurement, and meaningfulness of the questionnaire items and calculated Cronbach's alpha to gauge the internal consistency of the 25 items in the CDI. A score obtained of 0.91 indicates a strong level of internal consistency, confirming the reliability and cohesion of the items [25].

### Ethical approval

The study was conducted at Mutah University's Faculty of Nursing and was approved by the Research Ethics Committee. All participants provided informed consent, and the questionnaire incorporated a statement that ensured anonymity, confidentiality, and the option to withdraw from the study.

### Procedure

Following the necessary permissions granted by the faculty to distribute surveys within classrooms for a duration of two weeks, students were approached to participate. The participants were encouraged to complete the questionnaires and ensure anonymity by depositing them in the researcher's mailbox located at the Faculty of Nursing.

### Statistical analysis

The data analysis in this study used the 17th version of the Statistical Software for Social Sciences (SPSS). To enter the analysis, mean scores were calculated for each specific domain using items from the CDI, which covers four distinct care-related domains. Furthermore, the study incorporated predefined thresholds for CDI that were initially outlined in the original investigation and were subsequently utilized in the present study for accurate reporting purposes [20].

## Results

The participants were 105 fourth-year nursing students with a mean age of 23.4 years (SD=1.4). Most of the participants were females (*n* = 80, 76.2%), while males represent (*n* = 25, 23.8 %) (see Table 1). The participants represented 76% of the fourth-year students, while 640 students were enrolled in all the years.

**Table 1:** The demographics of the study participants

Demographic item		Respondents of Fourth-year students No. (%)	Total students in the school
Gender	Male	25 (23.8%)	
	Female	80 (76.2%)	
Age (years)	Minimum	21	
	Maximum	28	
	Age Mean	23.4	
	Std. Deviation	1.4	
Nursing Program	Regular	82 (78.1%)	489
	Parallel	17 (16.2%)	76
	Bridging	6 (5.7%)	62
	International	0 (0%)	3
	Total	105 (100%)	640

### Level of Psychosocial Care

Fourth-year nursing students showed significantly elevated average scores in the realm of professional and technical care, with a mean score of 4.69 ± 0.25, as indicated in Table 2. A detailed examination of the table demonstrates that all items associated with professional and technical care domains yielded remarkably high mean scores, ranging from (4.39 ± 0.90) to (4.84 ± 0.38).

**Table 2:** The mean scores and standard deviations of the participants’ responses on the CDI

**Professional and technical domain**		
** Items**	**Mean**	**SD**
Measurement of vital signs of a patient	4.84	0.38
Making a nursing record about the patient	4.79	0.51
Technically competent with a clinical procedure	4.76	0.52
Organizing the work of others for a patient	4.75	0.60
Observing the effects of a medication on a patient	4.75	0.49
Reporting a patient condition to a senior nurse	4.7	0.53
Consultation with a doctor about the patient	4.7	0.55
Having a neat dress when working with a patient	4.5	0.79
Explaining clinical procedures to a patient	4.39	0.90
**Psychosocial domain**		
** Items**	**Mean**	**SD**
Providing privacy for a patient	3.76	1.34
Listening to a patient	3.63	1.64
Providing reassurance about a clinical procedure	3.55	1.32
Assisting patient with an activity of daily living	3.48	1.38
Sitting with a patient	3.47	1.19
Exploring a patient life style	3.41	1.25
Being honest with a patient	3.3	1.53
Involving a patient with his or her care	3.29	1.46
Getting to know the patient as a person	3.28	1.39
Instructing a patient about an aspect of self-care	3.28	1.50
Being with a patient during clinical procedure	3.14	1.32
Keeping relatives informed about a patient	2.83	1.20
**Altruism (Appropriate self-giving) domain**		
** Items**	**Mean**	**SD**
Being cheerful with a patient	3.76	1.22
Putting the needs of a patient before your own	3.35	1.42
**Inappropriate (self giving) involvement domain**		
** Items**	**Mean**	**SD**
Sharing your personal problems with a patient	2.39	1.36
Feeling sorry for a patient	2.94	1.44

On the contrary, the average score for psychosocial care practices among participants was recorded at 3.37 (SD = 0.95). This score signifies a relatively lower level of psychosocial care exhibited by the participants compared to their notable achievements in professional and technical care. (see Table 2).

The item with the highest mean score (M=3.76, SD=1.34) was "Providing privacy for a patient." While the item "Keeping relatives informed about a patient" had the lowest mean score (M=2.85, SD=1.20) (see Table 2).

In the context of nursing education in the institution under study, it has been identified that although significant attention is directed toward technical and professional aspects, there is a notable need for nursing students to develop psychosocial competencies prior to graduation. These skills play a crucial role in enhancing students' abilities to apply them in their nursing interventions. Acquiring psychosocial skills equips nursing students with the necessary tools to excel in various dimensions of patient care.

## Discussion

The CDI encompasses 12 elements pertaining to psychosocial aspects. This study found that the average scores for this particular aspect were significantly lower than those for the professional and technical aspects. This discrepancy highlights the preferences of nursing students in Jordan for nursing care. The participants appeared to value technical care above psychosocial care. This suggests that their perception of care is molded over the course of their theoretical and practical studies, culminating in the completion of their nursing education. Furthermore, their adoption of professional behaviors is also influenced by their interactions with registered nurses in their field of practice, shaping their professional socialization [26]. Watson and his colleagues [27] also claimed that after finishing nursing education, nursing students tend to lose some of their idealistic views about nursing and caring. Safadi and her colleagues [28] also discussed the theory-practice gap and how student perspectives changed over the course of the educational process. They stressed the importance of updating the Jordanian nursing school curricula and university admission procedures [28].

Fourth-year nursing students are more likely to focus on technical and professional care at the expense of the psychosocial domain [16]. This could be related to a flawed educational system [27]. Students were often asked to do paperwork and assignments focusing on specific course outcomes, such as writing nursing care plans, seminars, and presentations about specific diseases or health conditions. They were also advised to perform specific nursing practices such as measuring vital signs, wound dressing, pre-preparation of medication, intravenous fluid, etc. All of these activities within their daily education and training routine emphasized the professional and technical aspects of care rather than engaging with the client in purposeful communication and spending time with clients for the purpose of the psychosocial aspect of care. This situation is accompanied by an overcrowded training environment owing to the high admission rate and limited hospital beds. Furthermore, the clinical setting in which they trained was a public hospital serving a large population with an extensive demand for services.

Early burnout may be predicted by poor psychosocial CDI item scores of fourth-year nursing students. Burnout has negative effects on academic performance, professional retention, and performance in the workplace [27, 29]. These results were expected with due attention. Previous surveys conducted at the same location revealed that students' satisfaction with the nursing program was indifferent and that one-third of them did not feel a sense of community within the nursing field [16, 30]. In addition, an earlier study conducted in Jordan on the beliefs, attitudes, and perceived practices of newly enrolled students corroborated this [31]. Only 31% of the students who participated in the survey at Jordanian nursing colleges and institutes affiliated with the Ministry of Health expressed a desire to become nurses. However, 69% of people started their nursing careers for other reasons, such as financial constraints from their families or because it was their only option to study [31].

### Limitations

This study has some limitations. First, the generalizability of the findings may be limited, because the study was conducted solely at a single university. Another limitation of this study is that the data collection relied on self-reported surveys, which could introduce potential bias due to respondents' tendency to provide socially desirable answers. This study did not assess the quality of psychosocial care provided by nursing students. The impact of psychosocial care on patient outcomes was not assessed in this study. These two limitations present opportunities for future research to explore them in greater depth.

### Implications

This study’s findings have several implications for nursing education and practice. First, nursing programs must prioritize the education and training of students in psychosocial care. This may include developing coursework and training programs focused specifically on psychosocial care as well as providing opportunities for hands-on training and simulation exercises.

Second, nursing practice settings must prioritize the provision of high-quality psychosocial care to patients. This may include providing resources and support for nurses to provide psychosocial care effectively as well as encouraging ongoing professional development and engagement with the latest research and best practices related to psychosocial care.

Finally, policymakers and healthcare organizations must prioritize the integration of psychosocial care into healthcare delivery systems. This may include developing policies and procedures that support the provision of psychosocial care as well as providing funding and resources for the training and education of healthcare professionals in this area.

## Conclusion

This study reported that nursing students perceive technical care to be more important than psychosocial care. These results indicate that psychosocial care for patients needs to be reoriented at multiple levels. This should include students, nursing schools, their curricula, training settings, clinical instructors, and educators. These nurses need to equip them with the competencies of holistic care for patients and their families, including psychosocial competencies, as a demand for their graduation from nursing programs. This is required at the time of the rapid complexities of healthcare delivery to achieve the essence of the nursing profession as a caring profession.

## Data Availability

All data generated or analyzed during this study are included in this published article.
